# Dietary Creatine and the Risk of Mental Health Conditions in the General Population

**DOI:** 10.1080/15502783.2025.2533644

**Published:** 2025-07-16

**Authors:** Dragana Zanini, Sonja Baltic, Sergej M. Ostojic

**Affiliations:** a University of Pécs, Faculty of Health Sciences, Pécs, Hungary; b University of Agder, Department of Nutrition and Public Health, Kristiansand, Norway; c University of Novi Sad, Applied Bioenergetics Lab, Faculty of Sport and PE, Novi Sad, Serbia

**Keywords:** Dietary creatine, mental health, depression, anxiety, suicide risk, nutrition, public health, nutraceuticals

## Abstract

**Background:**

Public health initiatives seek effective, affordable, and safe adjunctive therapeutics for treating mental health disorders. Preliminary studies have suggested that creatine may function as a nutraceutical agent with antidepressant-like effects.

**Methods:**

Data were extracted from the 2022 Korean National Health and Nutrition Examination Survey (KNHANES) for 5,257 individuals aged 12 years and older (56% female, mean age 51.1 ± 19.0 years) who reported dietary creatine intake and had an assessment for at least one mental health condition.

**Results:**

Mean daily creatine intake significantly differed between individuals with and without depression (8.16 ± 8.24 mg/kg body mass vs. 10.17 ± 9.34 mg/kg body mass; P = 0.002). One-way ANOVA revealed a significant difference in depression scores across quartiles of creatine intake (F = 4.003, P = 0.007), with individuals in the lowest quartile exhibiting significantly higher depression scores than those in all other quartiles (P ≤ 0.05). The prevalence of depression was highest among respondents in the lowest quartile of dietary creatine intake (6.9%) compared to those in the second (3.3%), third (4.3%), and fourth quartiles (3.6%) (P ≤ 0.05). A transition from the lowest to the highest quartile of daily creatine intake was associated with a 0.13-point reduction in depression scores. Participants in the lowest quartile also had significantly higher rates of suicidal thoughts, suicide planning, and suicide attempts compared to those in higher quartiles (P ≤ 0.05). Higher creatine intake was associated with a reduced risk of depression (OR 0.83, P = 0.010) and generalized anxiety disorder (OR 0.88, P = 0.041).

**Conclusion:**

Consistent with previous research, our findings suggest that dietary creatine intake may have modest beneficial effects on mental health. However, these results should be interpreted with caution, and further studies are required to validate these associations.

